# Effect of Alkyl Peroxyl Radical Oxidation on the Oxidative Stability of Walnut Protein Emulsions and Their Adsorbed Proteins

**DOI:** 10.3390/foods13101513

**Published:** 2024-05-13

**Authors:** Xue Wang, Qingzhi Wu, Xiaoying Mao, Jian Zhang

**Affiliations:** School of Food Science and Technology, Shihezi University, Shihezi 832003, China

**Keywords:** walnut protein emulsion, AAPH oxidation, oxidative stability

## Abstract

Walnuts are high in protein content and rich in nutrients and are susceptible to oxidation during production and processing, leading to a decrease in the stability of walnut protein emulsions. In this paper, the effect of alkyl peroxyl radical oxidation on the stability of walnut protein emulsions is investigated. With the increase of 2,2-azobis (2-methylpropionamidine) dihydrochloride (AAPH) concentration, both its protein and fat were oxidized to different degrees, and the droplets of the emulsion were first dispersed and then aggregated as seen from the laser confocal, and the stability of walnut protein emulsion was best at the AAPH concentration of 0.2 mmol/L. In addition to this, the adsorption rate of adsorbed proteins showed a decreasing and then an increasing trend with the increase in the oxidized concentration. The results showed that moderate oxidation (AAPH concentration: 0–0.2 mmol/L) promoted an increase in protein flexibility and a decrease in the protein interfacial tension, leading to the decrease in emulsion droplet size and the increase of walnut protein emulsion stability, and excessive oxidation (AAPH concentration: 1–25 mmmol/L) weakened protein flexibility and electrostatic repulsion, making the walnut protein emulsion less stable. The results of this study provide theoretical references for the quality control of walnut protein emulsions.

## 1. Introduction

Walnuts are an excellent source of high-energy food [1] which is native to southeastern Europe, East Asia, and North America. Walnuts are the most widely cultivated nuts in the world and were introduced to China during the Han Dynasty, with a history of cultivation of more than 1700 years, with China currently being the world’s top producer of walnuts. In the Chinese market, in addition to direct consumption, walnuts can also be used to extract walnut oil [2] and processed into walnut products, such as iodized oil, cooking oil, walnut yogurt, etc. [3].

During the production of walnut oil, a significant quantity of byproducts, known as walnut meal [4], is generated. This byproduct has a protein content ranging from 50% to 60% [5]. Unfortunately, it is currently either utilized as animal feed or simply discarded, leading to a wastage of resources. Regrettably, consumers have not shown sufficient interest in this matter. In order to utilize high-value walnut byproducts, walnut meal is often used as an important source of walnut protein. Walnut meal still contains residual lipids after walnut oil extraction, and residual lipids easily cause the oxidation of protein in the defatted walnut meal [6], resulting in changes in the structure and stability of proteins. Consequently, this has a definite effect on the healthful power of walnut protein.

The chemical compound 2,2-azobis (2-methylpropionamidine) dihydrochloride (AAPH) is a widely recognized radical initiator that is soluble in water and effectively initiates the reactions of lipid peroxidation [7]. Under aerobic conditions, it is able to pyrolyze to produce carbon-centered alkyl peroxyl radicals, which have the ability to modify lipids and other substances [8]. Lipid peroxidation is a non-enzymatic reaction that occurs spontaneously and involves a chain reaction. The peroxyl radical (ROO-) plays a crucial role as the main species in this chain reaction. The ROO- reaction facilitates the propagation of protein chain oxidation by abstracting hydrogen elements from the diallyl ethylene groups present in polyunsaturated fatty acids [9]. The process of protein oxidation in ROO- has a profound impact on the structural and chemical modifications of proteins, ultimately resulting in functional changes. Wu Wei [10], Ye Lin [11], and Zhou Feibai [12] have used AAPH thermal degradation under aerobic conditions to generate alkyl peroxyl radicals to oxidize soybean, peanut, and pork proteins. The studies have revealed that alkyl peroxyl radicals have a notable impact on the structure and characteristics of these proteins. Oxidized proteins demonstrate numerous improved functional characteristics, such as increased foaming, emulsification, and gelation capabilities. Zhu Zengfang [13] discovered that the process of AAPH oxidation can modify the protein structure of chickpea isolates. Furthermore, proteins that were excessively oxidized (with a concentration of 25 mmol/L AAPH) exhibited enhanced ability to produce foam, but their foam stability decreased due to the creation of insoluble aggregates.

Presently, the majority of research on walnut proteins primarily examines the extraction process [14,15] and the structural arrangement of walnut proteins [16,17,18]. In recent years, a few studies have also investigated the ability of walnut proteins to form emulsions [19]. Nevertheless, there is a lack of research regarding the long-term stability of walnut protein emulsion and the impact of protein oxidation on the adsorbed protein. Hence, this study employed AAPH as an oxidizing agent to replicate the authentic lipid peroxidation process. Consequently, the walnut protein emulsion underwent oxidative modification through the action of peroxyl radicals produced via the thermal decomposition of AAPH. The aim of this experiment is to examine the degree of protein oxidation and lipid oxidation in walnut protein emulsions under different levels of oxidation. Additionally, the study seeks to investigate the influence of various oxidation degrees on the emulsification capabilities of emulsions made from walnut protein. The results of this study will enhance the establishment of a theoretical basis and offer significant perspectives for quality assurance procedures in the production and preservation of walnut protein products.

## 2. Materials and Methods

### 2.1. Materials

Xinjiang 185 thin-skinned walnuts were purchased from Xinjiang Shihezi City Farmer’s Market (Shihezi, China), AAPH (2,2′-Azobis(2-amidinopropane) dihydrochloride) was purchased from Wako Pure Pharmaceuticals Co. in Osaka, Japan, DNTB (5,5′-dithiobis(dinitrobenzoic) acid salt) was purchased from Shanghai Yuanye Biotechnology Co., Ltd. (Shanghai, China), ethylenediaminetetraacetic acid (EDTA) was purchased from Shanghai Jinjinle Industry Co., Ltd. (Shanghai, China), and sodium dodecyl sulfate (SDS) was purchased from Tianjin Kaitong Chemical Reagent Co. (Tianjin, China). All other chemicals were of analytical reagent grade and were produced in China.

### 2.2. Preparation of Walnut Protein

With reference to the method of SZE-TAO K W C [20], although slightly modified, fresh walnuts were crushed after shelling and peeling and hexane was added and stirred at a ratio of 1:5 (*w*/*v*) for 1 h. Then, filtration was carried out, the residue was collected, and the previous operation was repeated until the filtrate became colorless and transparent. The residue was put into a fume hood to be dried up and, after completing the above steps, sieving was carried out to obtain defatted walnut powder. Then, the methods of alkaline dissolution and acid precipitation were used to extract the walnut protein. Finally, the walnut isolate protein were placed in the freeze dryer at 4 °C for freezing. The protein content was measured as 86.53% by Kjeldahl (SKD-3000, Xi’an Yupu Instrument Co., Xi’an, China) [21].

### 2.3. Preparation of Oxidized Walnut Protein

Referring to the method of Zhu Zengfang [13], the walnut had a protein content of 10 mg/mL, with 0.5 mg/mL of NaN_3_, after melting the protein in a 0.01 mol/L pH 8.0 phosphate buffer. AAPH (2,2′-azino-bis(2-amidinopropane) dihydrochloride) was added to the protein solution to produce AAPH concentrations of 0 mmol/L, 0.04 mmol/L, 0.2 mmol/L, 1 mmol/L, 5 mmol/L, and 25 mmol/L. On a shaker with a black hood, the designed protein solution was made to react for 24 h at 37 °C. After being removed, it was rapidly iced to 4 °C and subjected to spinning for 15 min at 10,000 rpm (at 4 °C) in order to eliminate any insoluble substances. It was dialyzed in deionized water for 72 h while being kept in a refrigerator at 4 °C. To get rid of unreacted AAPH, deionized water was changed every six hours. In order to obtain oxidized walnut protein, it was finally dried using the freeze drying method and stored for later use at 4 °C in the refrigerator.

### 2.4. Secondary Structure

Fourier transform infrared spectroscopy (FTIR) (IRXross, Shimadu, Kyoto, Japan) was used to determine the secondary structure of proteins using the Yan [22] et al. approach. An infrared spectrometer was used to perform full-band scans (4000–400 cm^−1^) at a resolution of 4 cm^−1^ for a total of 32 scans. The samples were mixed with 100 mg of potassium bromide powder, formed into tablets, and pressed in a tablet press after being thoroughly ground using an onyx mortar.

### 2.5. Contact Angle

Although slightly modified, the contact angle was calculated using Li et al. [23] as a guide. An infrared tablet press was used to press a suitable amount of sample powder into a mold. Water droplets were introduced using an autosampler to the pressed samples, which were set on a bench. Digital camera photos of the water droplets were acquired, and the anthropomorphic technique was used to calculate the contact angle.

### 2.6. Preparation of Emulsion

The measurement method by Li [24] was referred to and slight modifications were made. The walnut protein, which was precisely measured, was dissolved in a phosphate buffer with a concentration of 10 mmol/L and a pH of 7.4. Corn oil was then added to achieve a protein concentration of 1 g/100 mL and a corn oil concentration of 10 g/100 mL. The above mixture was pre-emulsified using a high-speed homogenizing blender (10,000× *g*, 2 min) and it was then sonicated using an ultrasonic cell crusher (300 w, 3 min). The freshly prepared emulsion was obtained.

### 2.7. Determination of Emulsion Carbonyl Content

The quantification of carbonyl content was conducted using the Levine method [25]: a volume of 4 mL of hydrochloric acid (HCl) with a concentration of 2 mol/L was combined with a solution containing 10 mmol/L of DNPH and added to the emulsion. The resulting mixture was then placed in a water bath at a temperature of 30 °C and left undisturbed for a duration of one hour in a dark environment. An equivalent volume of the emulsion was combined with 4 mL of 2 mol/L HCl without DNPH to create the blank condition, which was likewise kept in the dark for one hour. The reaction was shaken every 10 min with a vortex shaker and placed in the dark. To halt the reaction, a 4 mL aliquot of a 20% TCA solution was introduced into the centrifuge tube. The contents were well mixed until homogeneity was achieved, followed by a 10 min incubation period. Subsequently, centrifugation was performed for 5 min at a force of 11,000× *g*. The supernatant was carefully poured out and the precipitate was washed three times by centrifugation with 4 mL of ethanol–ethyl acetate solution (1:1, *v*/*v*). The precipitate was dissolved by agitating it in a solution of 6 mol/L guanidine hydrochloride (20 mmol/L KH_2_PO_4_, pH 2.3), maintained at a temperature of 30 ± 2 °C for a duration of 15 min. Subsequently, it was subjected to centrifugation at a force of 11,000× *g* for a period of 10 min and the supernatant was retained. The absorbance at 370 nm was detected using a UV–visible spectrophotometer (Cary50, Shanghai Spectrum Instrument Co., Ltd., Shanghai, China).

### 2.8. Measurement of Emulsion Sulfhydryl Content

The measurement method of Berton [26] was referred to and slight modifications were made. Preparation of buffer 1: 10.4 g Tris, 6.9 g Gly, 1.2 g EDTA, dissolved in deionized water and having a volume of 1 L and an adjusted pH of 8.0; Ellman’s reagent preparation: accurately weighed 0.2 g DTNB was dissolved in 50 mL buffer 1; preparation of buffer 2: 480 g of urea was added to buffer 1 and the pH adjusted to 8.0. The samples were diluted 20-fold with 10 mM phosphate buffer, and 1 mL was mixed with 2 mL of Tris–Gly and 8 mol/L of the urea buffer 2 (pH 8.0) using a pipette gun for precision pipetting and 0.02 mL of Ellman’s reagent was added. The resulting mixture was vortex shaken and made to react at room temperature for 30 min. Subsequently, the absorbance at 370 nm was measured using a UV–visible spectrophotometer (Cary50, Shanghai Spectrum Instrument Co., Ltd., Shanghai, China). The concentration of mercaptan was determined by utilizing a molar extinction coefficient of 13,600 M^−1^cm^−1^. No samples were added to the blank control group.

### 2.9. Determination of Emulsion Surface Hydrophobicity

A total of 0.5 g of the emulsion and 2 mmol/L of the sodium phosphate solution (pH 7.0) were mixed, diluted to 2 mL, and 200 μL of 1 mg/mL bromophenol blue indicator solution was added. The solution was stirred at 25 ± 2 °C for 10 min, centrifuged at 1000× *g* for 15 min at 4 °C, the supernatant was diluted five-fold, and the absorbance value was measured at 595 nm using a UV–visible spectrophotometer and recorded as A_sample_. The phosphate solution with the addition of bromophenol blue was taken as the control and written down as A_control_ [27].

(1) $$BBB(\mu g)=\frac{200\times (A_{control}-A_{sample})}{A_{contral}}$$

where BBB refers to Bromophenol Blue Binding (μg) and A refers to the absorbance.

### 2.10. Emulsion Tryptophan Fluorescence Measurement

Using a fluorescence spectrophotometer (Lumilux 5100, Hing Wo Instrument Co., Shanghai, China), the emulsion (100 μL) was combined with PBS (9 mL, 10 mM, pH 7.0) to determine the fluorescence emission spectra. The wavelength of excitation, range of emission, slit width, and scanning speed were 283, 300–500, 0.5 nm, and 600 nm/min, respectively [28].

### 2.11. Determination of the Hydroperoxide Value of Emulsions

In freshly prepared emulsions, 0.02% NaN_3_ was added to prevent microbial action. It was then immediately dispensed into centrifuge tubes and incubated in an electrically heated incubator at 50 °C for 17 days to promote oxidation reactions, and a small amount of sample was removed from the dispensed samples every other day for determination of the oxidation experiments.

For the determination of lipid peroxidation, we refer to the method of Shantha [29]: a centrifuge tube was filled with 0.3 mL of emulsion, 1.5 mL of a 3:1 (*v*/*v*) mixture of iso-octane and isopropanol, and the mixture was thoroughly shaken (10 s, three times). Subsequently, the combination underwent centrifugation at a force of 1000 g for a duration of 2 min. Following centrifugation, 200 μL of the chemical layer, which corresponds to the uppermost layer of the supernatant, was removed. The organic layer (upper layer) consisted of 200 μL, which was introduced into 2.8 mL of a 2:1 (*v*/*v*) mixture of methanol and butanol. Afterward, a total of 15 μL of a solution containing divalent ferrous ions (0.132 M barium chloride and 0.144 M ferrous sulphate combined in a 1:1 ratio) was mixed with 15 μL of ammonium thiocyanate after passing through a 0.22 μm filter membrane. After 20 min of reaction, the absorbance value at 510 nm was determined using a UV–visible spectrophotometer. Using the isopropyl benzene hydroperoxide reference curve, the concentration of peroxide in the sample was determined.

### 2.12. Measurement of Emulsion Malondialdehyde Products

The thiobarbituric acid (TBARS) values were established based on the method by Zhang [30]. A total of 1 mL of oleosome emulsion was mixed with 2 mL of a thiobarbituric acid test solution, which consisted of 0.375% thiobarbituric acid, 0.25 mol/L hydrochloric acid, and 15% trichloroacetic acid. The mixture was then subjected to a boiling water bath for 30 min. This was immediately followed by an ice bath for 10 min. Using a UV–visible spectrophotometer, the absorbance value at 532 nm was measured after the solution was run through a 0.22 μm filter membrane. The compound 1,1,3,3-tetraethoxypropane was employed as the reference substance to generate a normal curve. The compound used was 1,1,3,3-tetraethoxypropane and a calibration curve was generated.

### 2.13. Potentiometric Determination of Emulsion

Referring to Lin Shan Shi [31], although with slight modifications, the emulsion was diluted by a factor of 100 using a 5 mM phosphate buffer solution with a pH of 7.0. The potential of the emulsion oil droplets was measured using a Malvern Zetasizer Nano ZS (ZEN 3600, Malvern Instruments Ltd., Malvern, UK) analyzer. The temperature was measured at 25 ± 8 °C, and three measurements were done for each sample, with the average value being calculated.

### 2.14. Emulsion Stability Determination

The stability of the emulsions was evaluated by measuring the particle size and polymer dispersity index (PDI) values of the emulsions after a period of storage at room temperature (22 °C) and refrigerated temperature (4 °C) [32].

### 2.15. Measurement of Emulsification Activity and Emulsion Stability

Referring to the measurement method by Wang Ning [33], although with slight modifications, the just-created emulsion was placed in a 10 mL beaker. Next, 10 µL of the newly made emulsion was removed from the bottom of the beaker, vortexed, and combined with 7 mL of 0.1% SDS solution. At 500 nm, the absorbance was measured using a UV–visible spectrophotometer; the result was A_0_, and the 0.1% SDS solution concentration was utilized as a blank control. After 180 min of repose, the procedure was repeated and the absorbance was noted as A_180_. Both emulsifying activity index (EAI) and emulsion stability index (ESI) were determined using Equations (2) and (3):

(2) $$EAI=\frac{2\times 2.303}{C\times \left(1-\varphi \right)\times 10^{4}}\times A_{0}\times dilution\;factor$$

(3) $$ESI\left(\%\right)=\frac{A_{180}}{A_{0}}\times 100$$

where A_0_ and A_180_ are the absorbance values of the emulsion at 0 min and 180 min, φ is the volume fraction of the oil phase (*v*/*v*) (φ = 0.1), and C is the protein concentration.

### 2.16. Measurement of the Centrifugal Stability Constant

Referring to the method of Liu [34] for measuring the centrifugal stability constant of emulsion, 2 mL of emulsion was placed in a centrifuge tube and centrifuged at 3000× *g* for 10 min, and 50 μL of each of the lower layer of the emulsion before and after centrifugation was taken and fixed in 25 mL volumetric flasks with deionized water. Water was used as the blank, and the absorbance was determined by a UV–visible spectrophotometer at 500 nm, and the values of the absorbance before and after centrifugation were recorded as A_0_ and A. The centrifugal stability of the emulsions was evaluated according to the following methods:

(4) $$K_{E}=\frac{A_{0}-A}{A_{0}}\times 100\%$$

where K_E_ is the stability constant of the emulsion, the smaller the value of K_E_, the less the dispersed droplets float up under centrifugal force, the more stable the emulsion is.

### 2.17. Determination of Physical Stability of Emulsions

A total of 10 mL of the newly prepared emulsion was poured into a glass sample bottle with a lid, and 0.02% (*w*/*v*) of sodium azide (NaN_3_) was added to inhibit the growth of microorganisms in the emulsion, which was stored at 25 ± 2 °C for 14 days to observe the changes in the appearance of the emulsion. Photographs were taken for record keeping.

### 2.18. Laser Confocal Measurement

The microstructure of the emulsion was determined using a Nikon laser confocal instrument with reference to the measurement method of Wang Ning [33] and colleagues. The mass fractions of Nile red and Nile blue in the dyes were 0.1% and 1%, respectively, and were mixed homogeneously and stored at 4 °C away from light. Nile blue was used to stain the aqueous phase and Nile red for the oil phase. To make the determination, 1 mL of emulsion required to be mixed thoroughly, 20 µL of Nile red and 25 µL of Nile blue was added, and the mixture was allowed to stand for 30 min to allow the staining process to occur.

After setting the scanning parameters, the scanning was started, and the droplet images were captured and stored in the hard disk of the computer. NIS-Elements software 5.21 for laser scanning confocal microscope was used for image analysis and data processing.

### 2.19. Isolation and Extraction of Adsorbed Proteins

A 10 mL centrifuge tube was filled with 8.0 mL of each freshly made emulsion, and the tube was centrifuged at 15,000× *g* for 60 min at 20 °C, in accordance with the measurements by Chen [35] and others. The cream phase, which contained mostly adsorbed proteins, was kept at the bottom of the centrifuge tube and used for more adsorbed protein extraction, while the serum phase, which contained mostly unadsorbed proteins, was promptly transferred to a different tube. Additionally, to eliminate scattered, microscopic oil droplets, the serum phase was centrifuged once more at 15,000× *g* for 30 min at 20 °C. It was then successively filtered through cellulose acetate filters with pore sizes of 0.45 and 0.20 microns.

A total of 1 mL of the previously mentioned in situ cream phase was resuspended in 7.0 mL of 0.5% Tween 20 solution and vortexed for 30 s. The suspension above was centrifuged at 15,000× *g* for 60 min at 20 °C, and the lower supernatant was then centrifuged once more under the same conditions. The lower supernatant was then filtered through cellulose acetate filters that were 0.45 µm and 0.20 µm in order, and 5.0 mL of the above filtrate was diluted four times using a phosphate buffer (10 mM, pH 7.0). This was then transferred to an ultrafiltration centrifuge tube and centrifuged at 6000× *g* for 45 min at 20 °C. Ultimately, every concentrated solution of adsorbed protein (~0.2 mL) was gathered and diluted to 1.0 mL in anticipation of further study.

### 2.20. Determination of Protein Adsorption Rate of Adsorbed Proteins in Walnut Protein Emulsions

Following the procedure described by Liang [36], the emulsion obtained after the initial centrifugation was extracted from the lower clear layer using a 1 mL disposable syringe. The extracted emulsion was then passed through a 0.45 μm needle-type filter, appropriately diluted, and the protein concentration of the resulting filtrate, referred to as CSER, was determined using the BCA method. The protein adsorption rate was determined using the following formula:

(5) $$AP\%=\left(C_{INI}-C_{SER}\right)\times 100/C_{INI}$$

where C_SER_ and C_INI_ stand for the protein concentration prior to emulsifying the oil droplets and the unadsorbed protein concentration in the transparent layer, respectively.

### 2.21. SDS-PAGE of Adsorbed Proteins in Walnut Protein Emulsion

Interfacial proteins were extracted and adjusted to the same concentration. Subsequently, 80 µL of sample solution was mixed with 20 µL of up-sampling buffer (5× reducing type) and boiled in boiling water for 5 min. A total of 25 µL of prepared sample was added to the gel (consisting of 5% polyacrylamide concentrate and 12% polyacrylamide separating gel) and subunit separation was performed using an electrophoresis system (XINPOWER-300/600, Shanghai Qinxiang Scientific Instrument Co., Shanghai, China) at 80 V and 120 V. After staining and destaining, gel images were obtained via an imaging system [37].

### 2.22. Determination of Particle Size of Adsorbed Proteins in Walnut Protein Emulsions

A total of 1 mL of the adsorbed protein solution was transferred to a square cuvette and particle size was measured using a Malvern laser particle sizer, which was determined in the same way as in Section 2.14 [32].

### 2.23. Determination of Zeta Potential of Adsorbed Proteins in Walnut Protein Emulsions

A protein solution with an equal concentration of adsorbed protein was injected into a cuvette that was folded and had electrodes at both ends. The cuvette was then placed into a controlled pool chamber at a temperature of 25 ± 8 °C. The movement speed of the charged droplets toward the oppositely charged electrodes was measured using a Malvern laser particle sizer [31].

### 2.24. Statistical Analysis

All experiments were measured for three times. Results are expressed as mean ± standard deviation. The data were analyzed using the Origin 2018 software to generate figures, while SSPS 26.0 was utilized to perform one-way ANOVA analysis and calculate the standard deviation using Duncan’s test (*p* < 0.05), and the differences were considered significant with *p* < 0.05.

## 3. Results and Discussion

### 3.1. Effect of AAPH Oxidation on the Secondary Structure of Walnut Proteins

**Figure 1:**
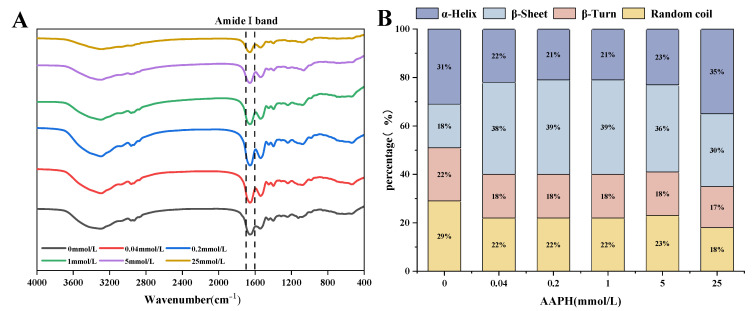
Effect of AAPH concentration on the secondary structure of walnut protein. Fourier transform infrared spectra (**A**) and secondary structure ratios (**B**) of walnut proteins at different oxidizing concentrations.

Fourier infrared spectroscopy is a technique that can be used to measure the structure of proteins in various physical states. The amide I band, which falls within the range of 1700–1600 cm^−1^, is a distinctive vibrational peak that corresponds to the protein secondary structure. This peak is commonly employed to detect alterations in the secondary structure of proteins. Figure 1A displays the FTIR spectra of walnut protein at various levels of oxidizing concentrations. As shown in Figure 1B, the ratio of secondary structure of walnut proteins at different oxidizing concentrations can be determined using the Peak-Fit version 4.04 software. The α-Helical structure is observed within the frequency range of 1646–1664 cm^−1^. The β-Sheet structure is detected within two frequency bands: 1615–1637 cm^−1^ and 1682–1700 cm^−1^. The β-Turn structure is found within the frequency range of 1664–1681 cm^−1^. The random coil structures are identified within the frequency range of 1637–1645 cm^−1^ [38].

Figure 1B reveals that the β-Sheet content in walnut protein increased at AAPH concentrations of 0.04 mmol/L, 0.2 mmol/L, and 1 mmol/L. Conversely, the content of β-Turn, random coil, and α-Helix remained relatively constant after a slight decrease at 0.04 mmol/L. This indicates that mild oxidation disrupts the secondary structure of walnut protein, while the stability of oxidized walnut protein remains largely unaffected. As the concentration of oxidation increases, the amount of β-Sheet decreases because β-Sheet has a relatively enlarged and structured structure. Therefore, the secondary structure of over-oxidized walnut protein becomes chaotic and loose, losing its original order [39].

### 3.2. Effect of AAPH Oxidation on the Contact Angle of Walnut Protein

**Figure 2:**
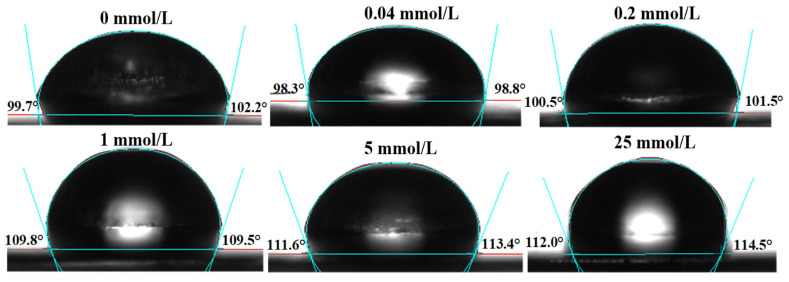
Effect of AAPH concentration on the protein contact angle of walnut.

Figure 2 displays the contact angle of walnut protein at various levels of oxidizing concentrations. The contact angle (θ) is a crucial characteristic for quantifying the surface wettability of a solid substance. When θ < 90°, the sample is considered to be hydrophilic, meaning that the surface of the solid material easily allows the liquid to wet it. The smaller the value of θ, the more hydrophilic the sample is. On the other hand, when θ > 90°, the surface of the solid material is considered to be hydrophobic. This means that the liquid does not easily wet the surface and tends to move on it instead. In this case, the larger the value of θ, the more hydrophobic the sample [40]. When the contact angle approaches 90°, the solid surface achieves hydrophilic/hydrophobic equilibrium, making it ideal for stabilizing emulsions [41]. Figure 2 demonstrates that, as the AAPH concentration increased from 0 mmol/L to 0.04 mmol/L, the θ value decreased and reached a minimum. This suggests that moderate oxidation enhanced the hydrophilicity of the walnut protein surface. However, as the AAPH concentration continued to increase, the θ values also increased, indicating that excessive oxidation made the surface of the solid walnut protein sample more hydrophobic. This is detrimental to the stability of walnut protein emulsions. This conclusion can be reaffirmed through the further analysis of particle size, potential, and laser confocal of walnut protein emulsions.

### 3.3. Effect of AAPH Oxidation on the Carbonyl and Free Sulfhydryl Content of Walnut Protein Emulsions

Important characteristics of protein oxidation include protein aggregation, an increase in carbonyl derivatives, and a decrease in protein sulfhydryl content. Protein oxidative modifications are changes in amino acid side chains or peptide chains brought on either directly or indirectly by reactive oxygen species. Hence, the identification of carbonyl compounds has been regarded as a significant parameter for characterizing proteins in terms of oxidation [42].

Table 1 illustrates how the carbonyl content of the walnut protein emulsion varies in the peroxyl radical oxidation system. The present study determined that the original carbonyl concentration of the walnut protein emulsion was 13 nmol/mg. The carbonyl content exhibited a 1.14-fold rise at an AAPH concentration of 1 mmol/L, and a 1.42-fold increase at an AAPH concentration of 25 mmol/L. The observed trend suggests a notable rise in the carbonyl content as the AAPH oxidizing concentration increases (*p* < 0.05). This suggests that the peroxyl radicals formed by the gradual increase in the concentration of AAPH lead to the gradual oxidation of the walnut protein emulsion. The formation of alkyl peroxyl radicals by thermal degradation of AAPH attacks amino acid residues [43] or oxidation leads to disassembly of the protein backbone [44], resulting in an increase in carbonyl content.

### 3.4. The Impact of AAPH Oxidation on the Concentration of Free Sulfhydryl Groups in Walnut Protein Emulsion

Research indicates that the primary source of some proteins’ antioxidant activity is their active sulfhydryl groups, which scavenge free radicals. However, as part of this process, the sulfhydryl groups themselves oxidize to form disulfides, hyposulfuric, sulfinic, or sulfonic acids [45]. Therefore, the measurement of sulfhydryl numbers can be an important indicator to characterize structural changes in proteins [46].

Table 1 illustrates the alterations in sulfhydryl concentration of walnut protein emulsion when subjected to peroxyl radical oxidation. The gradual increase in AAPH concentration resulted in a corresponding decrease in the free hydrophobic content of walnut protein emulsions. Specifically, the hydrophobic content decreased from 72.36 nmol/mg to 68.82 nmol/mg and further to 61.69 nmol/mg. At lower AAPH concentrations (0–0.2 mmol/L), the sulfhydryl content of the emulsions did not decrease significantly (*p* > 0.05), and the sulfhydryl content in general appeared to be gradually decreasing with the gradual increase of AAPH concentration. The phenomenon under observation exhibits similarities to the AAPH-induced oxidation process of soy protein [47]. The structural conformation of proteins is influenced by the presence of cysteine and disulfide bonds [48]. In the peroxyl radical oxidation system, peroxyl radicals initially interact with sulfhydryl groups, leading to the formation of sulfinyl radicals. These sulfinyl radicals subsequently combine with molecular oxygen to generate thiol radicals. The thiol radicals then undergo further oxidation, resulting in the formation of disulfide bonds and consequently promoting protein oxidation [49]. Gardner’s study [50] on the effect of cysteine and lipoprotein oxidation on the structure and emulsification properties of walnut proteins showed that lipid hydroperoxides form stable adducts with cysteine residues in proteins, which results in a decrease in the free sulfhydryl content of the protein. The results of this experiment showed that alkyl peroxyl radicals caused a fuller oxidation of walnut isolate protein, resulting in a significant decrease in the free sulfhydryl content within the protein molecule (*p* < 0.05).

### 3.5. Effect of AAPH Oxidation on the Surface Hydrophobicity of Walnut Protein Emulsion

The measurement of protein surface hydrophobicity functions as a measure of the number of hydrophobic molecules available on the protein surface. This measurement is employed to assess the occurrence of oxidation [51] and to gauge the extent of protein structure unfolding [52]. In the process of emulsion formation, the presence of hydrophobic groups promotes the development of a compact protein film on the lipid surface, resulting in enhanced emulsification capabilities of the emulsions [53]. The binding of bromophenol blue to the hydrophobic regions of proteins enables the quantification of surface hydrophobicity by assessing the extent of bromophenol-blue–protein interactions. An increased level of bromophenol blue binding is indicative of an elevated degree of surface hydrophobicity.

**Figure 3:**
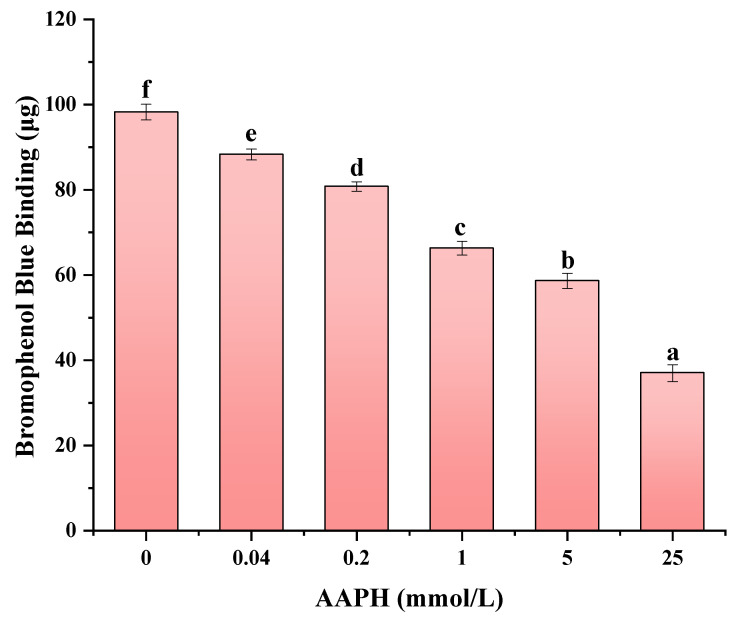
The impact of varying AAPH concentrations on the surface hydrophobicity of walnut protein emulsion. Values in a column followed by different letters are significantly different (*p* < 0.05).

As depicted in Figure 3, there is a noticeable decrease in the binding of bromophenol blue as the concentration of the oxidizing agent increases (*p* < 0.05). The aforementioned data suggest that there is a concurrent reduction in the surface hydrophobicity of the emulsion as the concentration of AAPH increases. At a concentration of 25 mmol/L, the AAPH compound induced a reduction of 62.35% in the affinity for binding of bromophenol blue. This decrease can be attributed to the capacity of peroxyl radicals to transform hydrophobic side-chain residues of proteins into hydrophilic moieties. Subsequent to their interaction with proteins, these hydrophilic entities have the capacity to engage in binding, thereby instigating the process of amino acid residue oxidation, as well as the unfolding and ensuing contact of hydrophobic groups [54]. As a result, the hydrophobic groups that are exposed have the tendency to come together, leading to a decrease in the hydrophobic nature of the surface. Numerous studies have provided evidence supporting the notion that the creation of protein aggregates is a result of surface hydrophobic interactions [55]. Zhu Zengfang [13] conducted a study which revealed that the spatial configuration of chickpea proteins experienced alterations when subjected to oxidation by AAPH. Furthermore, the study demonstrated that elevated levels of oxidation facilitated the aggregation of proteins and the production of hydrophilic groups. Protein aggregation involves the process of sequestering or encapsulating hydrophobic regions, thus retaining soluble aggregates that exhibit reduced surface hydrophobicity and ultimately leading to a decrease in protein surface hydrophobicity.

### 3.6. Effect of AAPH Oxidation on Tryptophan Fluorescence in Walnut Protein Emulsion

**Figure 4:**
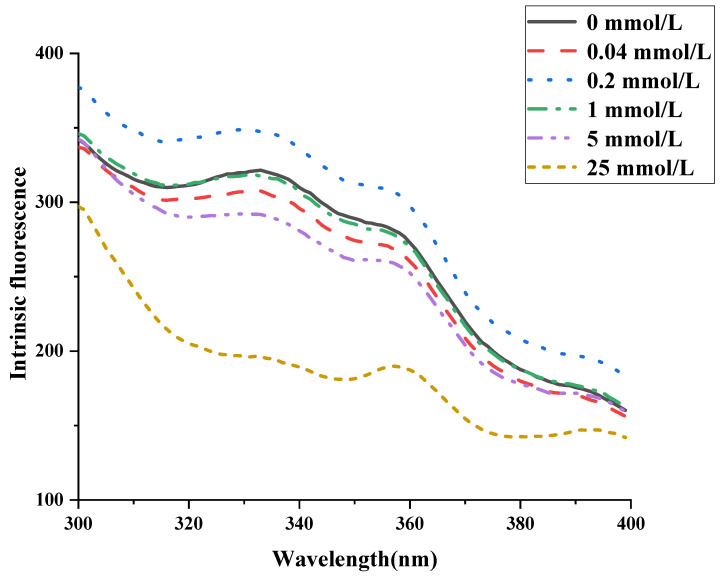
Effect of AAPH concentration on the intrinsic fluorescence of walnut protein emulsion.

Endogenous fluorescence can reflect the degree of oxidation of protein tryptophan residues and changes in the microenvironment to characterize the effect of oxidation on protein tertiary structure [13]. Figure 4 displays the observed fluctuations in endogenous fluorescence intensity of walnut protein emulsions. It is noteworthy that the emulsion containing no AAPH addition had a maximum fluorescence peak position at 333 nm. The value of λ max is influenced by the microenvironment surrounding the tryptophan residue. A λ max value below 330 nm suggests that the tryptophan residue is situated within the nonpolar interior of the protein molecule. Conversely, a λ_max_ greater than 330 nm indicates that the tryptophan residue is located in a polar environment external to the protein molecule. A slight redshift in the maximum wavelength of endogenous fluorescence occurred when the walnut protein emulsion was oxidized by low concentrations of AAPH, suggesting that tryptophan residues move closer to the polar microenvironment during protein defolding. However, the maximum wavelength of endogenous fluorescence, denoted as λ max, began to decrease once more and experienced a blue shift when the concentration of AAPH exceeded 1 mmol/L. This suggests that the tryptophan residue groups during protein aggregation are located in a more hydrophobic (nonpolar) microenvironment. This is consistent with the trend of endogenous fluorescence change in peanut isolate protein oxidized by peroxyl radicals reported by Ye [11]. With an increase in the concentration of AAPH, there was a modest rise in fluorescence intensity observed at an AAPH content of 0.2 mmol/L, followed by a subsequent decrease. The reason for this is that tryptophan possesses the lowest one-electron oxidation potential compared to the other 19 amino acids found in proteins. AAPH oxidation converts Try to kynurenine in proteins, encapsulating the previously exposed tryptophan residues [56], resulting in a decrease in endogenous fluorescence intensity. The oxidation of proteins in emulsions by AAPH was confirmed through the analysis of the carbonyl group, sulfhydryl group, surface hydrophobicity, and tryptophan fluorescence.

### 3.7. Lipid Oxidation in Walnut Protein Emulsion

The oxidation process of lipids in emulsions serves as a significant determinant of the quality of emulsion-based food products. In oil-in-water emulsions, the oxidation of lipids predominantly takes place at the interface between the oil and the water phases. This occurrence is attributed to the interaction between pro-oxidants, including free radicals and transition metals present in the continuous phase, and hydroperoxides located on the surface of the emulsion droplets. Consequently, this interaction leads to the oxidative degradation of the lipids, resulting in their deterioration [57].

**Figure 5:**
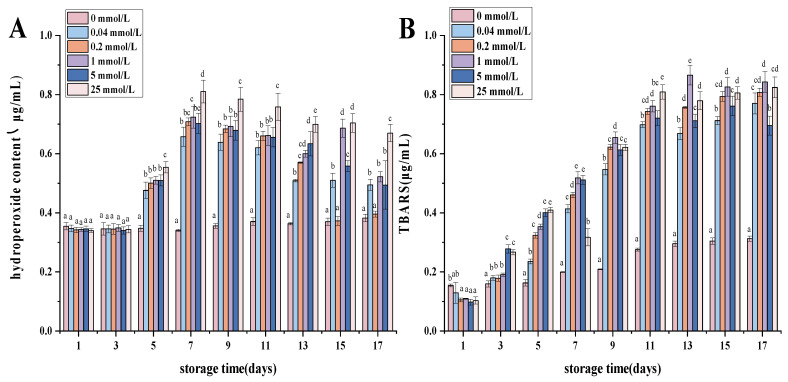
Lipid oxidation of walnut protein emulsion stored at 50 °C for 17 days. All six samples were stored for 17 days. (**A**) lipid hydrogen peroxide value; (**B**) TBARS content. The values of different letter columns were significantly different (*p* < 0.05).

The assessment of oxidative stability in walnut protein emulsions during storage can be accomplished by quantifying the primary products of lipid oxidation, namely hydroperoxides, as well as the secondary products, such as TBARS [58]. The levels of hydroperoxides and TBARS in all fluids exhibited a progressive increase over time, suggesting that the oils underwent oxidation during the storage period [22]. Figure 5 depicts the assessment of the oxidative stability of walnut protein emulsions by tracking the principal oxidation products of the oils over a storage period of 17 days. In the AAPH oxidation system, the hydroperoxide content of fresh emulsions continued to increase throughout the pre-storage period and reached a maximum on the seventh day, indicating the occurrence of lipid oxidation. The hydroperoxide values decreased again with a further increase in storage time. This was mainly due to the conversion of some primary oxidation products into secondary oxidation products [59]. This assertion is substantiated by the significant increase in secondary oxidation byproducts seen during the storage period, as illustrated in Figure 5B (*p* < 0.05). The observed conditions can be ascribed to the interplay between proteins that have adsorbed onto the surface of the oil droplets and secondary reaction products, specifically aldehydes, which are generated due to lipid oxidation. The aforementioned processes have the potential to disrupt the emulsion’s lipid oxidation process or modify the functional characteristics of the proteins, resulting in a potential underestimation of the true extent of oxidation [57].

The degree of fat oxidation in emulsions was determined by measuring lipid oxidation secondary products (TBARS) in response to the oxidative stability of emulsions [60]. According to the data presented in Figure 5B, the malondialdehyde content in AAPH emulsions of different concentrations showed an increasing trend with increasing storage time. This may be due to the decomposition of the initial oxidation products with time, resulting in an increase in TBARS content [33]. The higher the AAPH concentration, the higher the malondialdehyde production, proving that the AAPH concentration is proportional to the rate of malondialdehyde production. Tong X [61] et al. found that the malondialdehyde content of enzymatically hydrolyzed soybean proteins for 15 days of storage increased with storage time.

### 3.8. Measurement of Emulsion Potential

**Figure 6:**
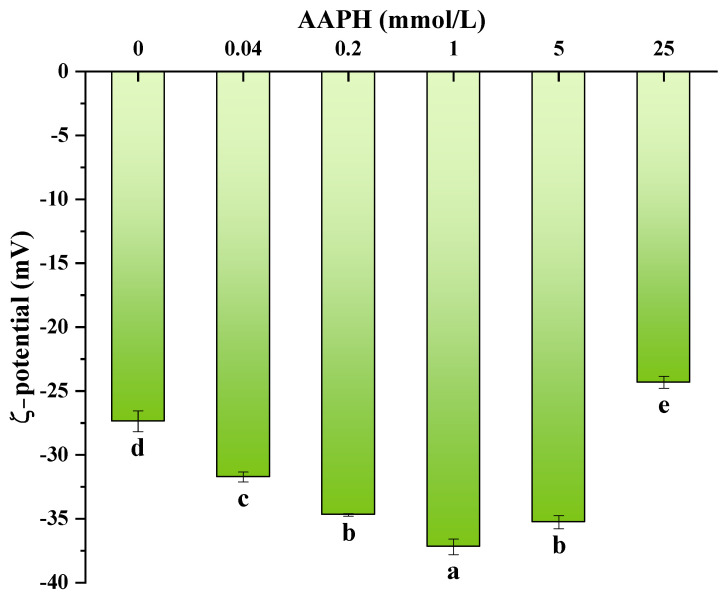
Effect of AAPH concentration on the zeta potential of walnut protein emulsion. Values in a column followed by different letters are significantly different (*p* < 0.05).

The zeta potential serves as a characterization of the extent of surface charge exhibited by an oil droplet [62]. Figure 6 displays the zeta potentials of emulsions containing walnut protein which have been subjected to varying degrees of oxidation. Notably, all emulsions exhibited negative zeta potentials. This result suggests that repulsive forces (mainly electrostatic and spatial) play a dominant role, and that attractive forces (mainly hydrophobic and van der Waals forces) are weak in the whole emulsion system [63]. The difference in zeta potential of proteins can reflect the degree of exposure of charged groups [64]. The zeta potential is a parameter that can be used to assess the stability of emulsions. A higher absolute value of zeta potential indicates a greater degree of stability in the system. Conversely, a reduction in stability leads to coagulation and aggregation of the solution [65]. In the alkyl peroxy radical oxidation system, it has been observed that the emulsion’s zeta potential reaches its maximum value when the concentration of AAPH is 1 mmol/L, this time corresponding to the best stability of the protein emulsion. With the progressive increase in the concentration of AAPH, a discernible reduction in the magnitude of the zeta potential was observed. This finding aligns with the outcomes observed in terms of emulsification activity and emulsion stability. According to Ye Lin [66], it has been observed that protein oxidation can result in a decline in the absolute level of potential in fresh emulsions.

### 3.9. Determination of Emulsion Stability

**Figure 7:**
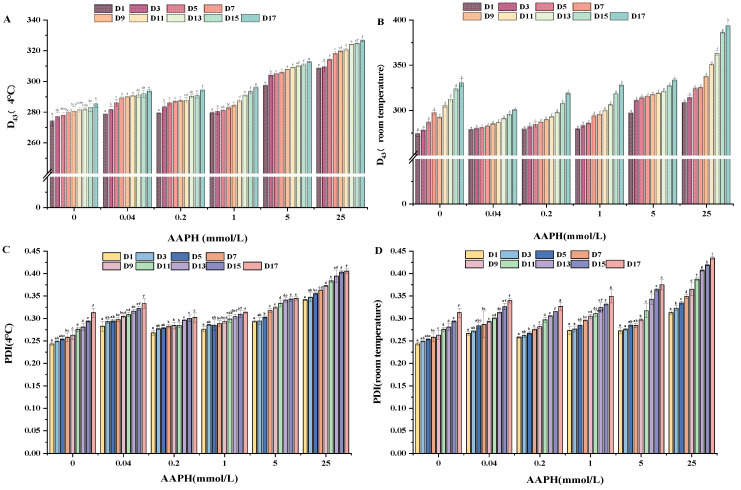
Effect of AAPH concentration on the stability of walnut protein emulsion. Particle size stored at 4 °C for 17 days (**A**), particle size stored at room temperature for 17 days (**B**), PDI value stored at 4 °C for 17 days (**C**), PDI value stored at room temperature for 17 days (**D**); different letter columns had significant differences on zeta potential of walnut protein milk (*p* < 0.05).

The particle size can directly reflect the aggregation state of proteins after oxidation with different AAPH concentrations. The particle size of AAPH oxidized walnut protein emulsion during 17 days of storage is shown in Figure 7A,B (4 °C and room temperature). The figure illustrates a positive correlation between the concentration of oxidation and the steady rise in particle size. The relationship between oxidation concentration and particle size has a positive correlation, whereby an increase in oxidation concentration leads to a corresponding increase in particle size. This may be due to the fact that oxidation causes the interfacial network structure to become less elastic and more viscous, which, in turn, destroys the interfacial network structure and increases the emulsion droplet size [67]. In addition, the particle size of emulsions stored at 4 °C was significantly lower than that of emulsions placed at room temperature. At room temperature, the particle size increased progressively as the storage time increased. During the initial storage period, the emulsion exhibited greater stability, with minimal changes in particle size. However, during the later stages of storage, particularly at room temperature, the particle size underwent more significant changes (*p* < 0.05). This can lead to the emulsion experiencing delamination and the formation of insoluble aggregates.

The PDI (polymer dispersity index) value serves as an indicator of droplet homogeneity. A lower PDI value corresponds to increased dispersibility and enhanced stability of emulsions. As illustrated in the diagram, it is apparent that the emulsion’s polydispersity index (PDI) was lower at a concentration of AAPH of 0.2 mmol/L in comparison to the other concentrations. This observation provides evidence to support the idea that moderate oxidation contributes to the improvement of emulsion stability. Conversely, as the concentration of AAPH increased, the PDI value exhibited a gradual increase, thereby diminishing the stability of the emulsion. Notably, on the 17th day of storage, when the AAPH concentration reached 25 mmol/L, the emulsion’s stability was at its lowest, resulting in significant delamination.

### 3.10. Determination of Emulsifying Activity and Stability

**Figure 8:**
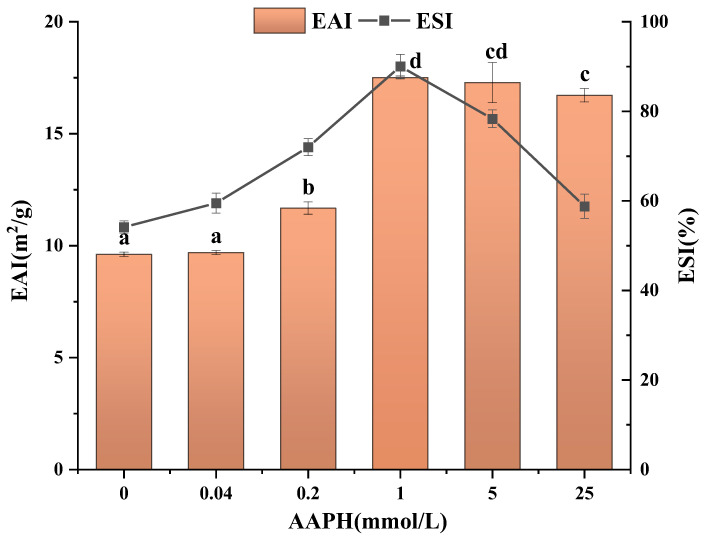
Effect of AAPH concentration on the emulsifying activity and stability of walnut protein emulsion. Values in a column followed by different letters are significantly different (*p* < 0.05).

The emulsion containing oxidized walnut protein is shown in Figure 8 together with its emulsification stability index (ESI) and emulsification activity index (EAI). The findings indicate that the alkyl peroxyl radical system, when subjected to AAPH oxidation concentrations of up to 1 mmol/L, resulted in walnut protein samples achieving an emulsification stability of 90.04% and a maximum emulsification activity index (EAI) value of 17.51 m^2^/g. The increase in emulsification capacity of walnut protein in an alkyl peroxyl radical oxidation system with lower oxidation concentration is due to the unfolding of the higher structure of the protein molecule by suitable oxidation, exposure of hydrophobic groups, adsorption of more proteins at the interface, and increase in the emulsification property of the protein. The increase in emulsification stability is due to the formation of disulfide bonds by the oxidation of sulfhydryl groups, resulting in covalent cross-linking between protein molecules and the formation of macromolecular aggregates that can form a more stable protein film on the surface of the oil droplet [68]. Therefore, moderate oxidation of proteins increases their emulsifying activity and emulsion stability. At high oxidizing concentrations, proteins are severely denatured, solubility decreases, aggregation occurs, and, ultimately, emulsifiability and emulsion stability decrease [69].

### 3.11. Determination of Emulsification Rate and Centrifugal Stability Constant

**Figure 9:**
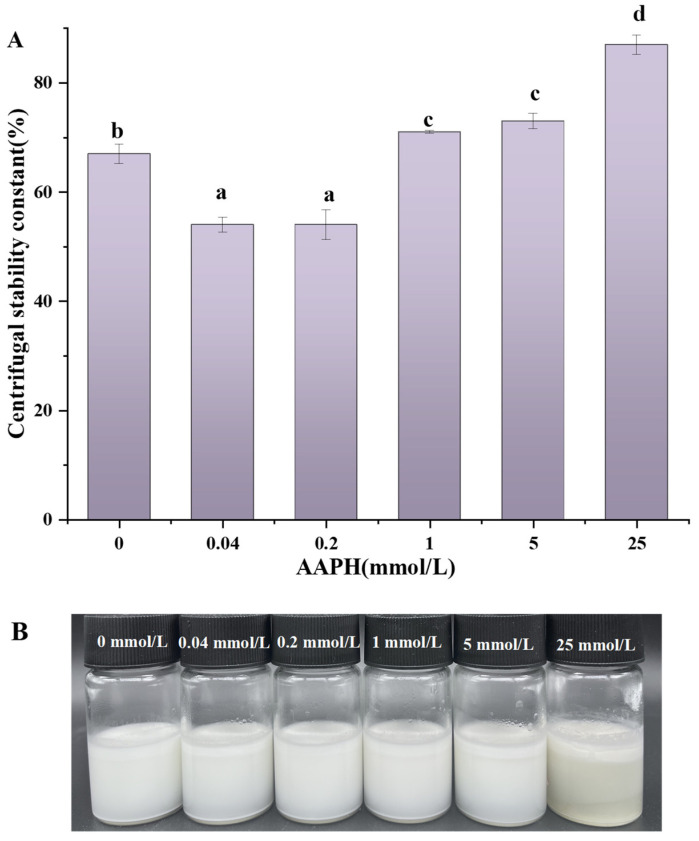
Effect of AAPH concentration on centrifugal stability constants of walnut protein emulsions (**A**) and walnut protein emulsions after 14 days of storage (**B**). Values in a column followed by different letters are significantly different (*p* < 0.05).

The centrifugal stability constant quantifies the emulsion capacity to maintain stability when subjected to centrifugal force. As shown in Figure 9A, the centrifugal stability constant exhibits a decreasing trend followed by an increasing trend as the oxidation concentration increases. This discovery suggests that the stability of emulsions can be improved through moderate oxidation, because this process reduces the likelihood of emulsion droplets to coalesce and form aggregate. As the oxidation concentration continues to rise, there is an observed increase in the stability constant of centrifugal force. This leads to the initiation of flocculation and agglomeration of emulsion droplets, ultimately resulting in a decreased emulsion stability due to the combined influence of gravity and centrifugal force. The findings of this study align with the outcomes observed in the context of AAPH-oxidized rice bran protein emulsion stability [24]. Figure 9B records the state of walnut protein emulsions after AAPH oxidation and 14 days of storage, from which it can be seen that, after 14 days of storage, emulsions with different AAPH concentrations were slightly delaminated, with emulsions being the least delaminated when the AAPH concentration was 0.2 mmol/L. As the oxidized concentration increased with the AAPH concentration of 1–25 mmol/L, the delamination of the emulsions also became more and more serious. Especially when the AAPH concentration was 25 mmol/L, the walnut protein emulsion had the most serious delamination compared to the emulsions with other oxidized concentrations. This is consistent with the results of the centrifugal stabilization constant.

Both results indicate that moderate oxidation stretches the protein spatial structure, enhances protein flexibility, and exposes hydrophobic groups buried within the protein [69], promoting protein adsorption at the oil–water interface and forming a tighter adsorption layer. Excessive oxidation has been observed to diminish protein flexibility and promote protein aggregation. This process involves the burial of sulfhydryl groups and electrically charged groups inside the protein structure, resulting in reduced protein adsorption. Consequently, the stability of the emulsion is compromised [70]. According to Fan Huan [71], it has been demonstrated that increased contacts between droplets contribute to the development of a gel network structure. This structure exhibits the ability to withstand specific mechanical pressures, thus improving the centrifugal stability of the emulsion. Consequently, this process results in the formation of a denser layer. The centrifugal stability of the emulsion results in an improved stability of the emulsion.

### 3.12. Laser Confocal Measurement

**Figure 10:**
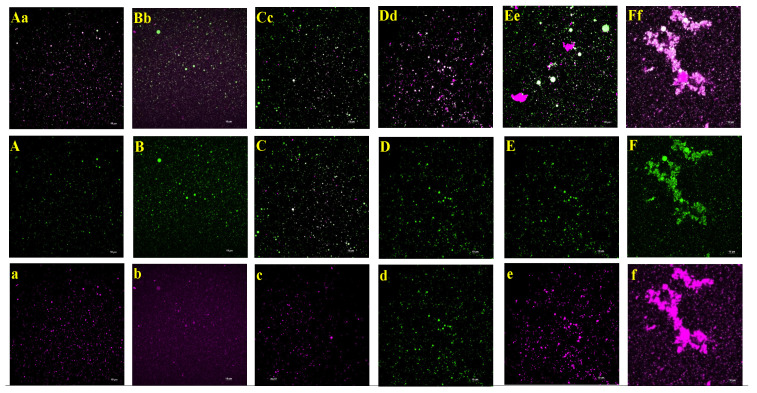
Effect of AAPH concentration on the microstructure of walnut protein emulsion. (**Aa**,**Bb**,**Cc**,**Dd**,**Ee**,**Ff**) represent the microscopic structure at 0 mmol/L, 0.04 mmol/L, 0.2 mmol/L, 1 mmol/L, 5 mmol/L, and 25 mmol/L oxidation concentration, respectively. Uppercase letters are protein staining and lowercase letters are oil droplet staining.

The emulsion droplet distribution was examined using laser scanning confocal microscopy. The provided Figure 10 depicts a distinct correlation between the concentration of AAPH and the particle size of the emulsion containing walnut protein. The emulsion particle size decreased as the concentration of AAPH boosted. Additionally, the interaction between the droplets, characterized by aggregation and flocculation, weakened as the AAPH concentration increased. The findings from the analysis of emulsion stability align with the observations made using optical microscopy and laser confocal microscopy. During the initial stage of oxidation (0–0.2 mmol/L), there was a gradual enhancement in the emulsifying activity and stability of the emulsion. This improvement in stability resulted in the dispersion of oil droplets and proteins throughout the emulsion. It is worth mentioning that the emulsion exhibited the most equal dispersion of oil droplets and proteins at an AAPH levels of 0.2 mmol/L. Nevertheless, as the concentration of AAPH was elevated, there was a noticeable rise in the size of emulsion droplets, leading to the formation of aggregates. This increase in aggregation was observed to occur gradually, as depicted in Figure 10Ff. Notably, at an AAPH concentration of 25 mmol/L, a substantial area of aggregation was formed by the oil droplets and proteins, expressing the highest degree of aggregation. It was demonstrated that excessive oxidation (1–25 mmol/L) caused flocculation and aggregation leading to poor emulsion stability, a conclusion which agrees with that obtained from the centrifugal force stability constant before. It is shown that moderate oxidation can serve to reduce emulsion droplet size and promote the formation of inter-droplet aggregates. One possible explanation is that moderate oxidation facilitates an augmentation in protein flexibility, reduction in protein interfacial tension, and reinforcement of spatial site resistance and electrostatic repulsion. Conversely, high levels of oxidation diminish protein flexibility, leading to an elevation in protein interfacial tension and a decrease in spatial site resistance and electrostatic repulsion [69].

### 3.13. Determination of Adsorption Rate of Adsorbed Proteins

**Figure 11:**
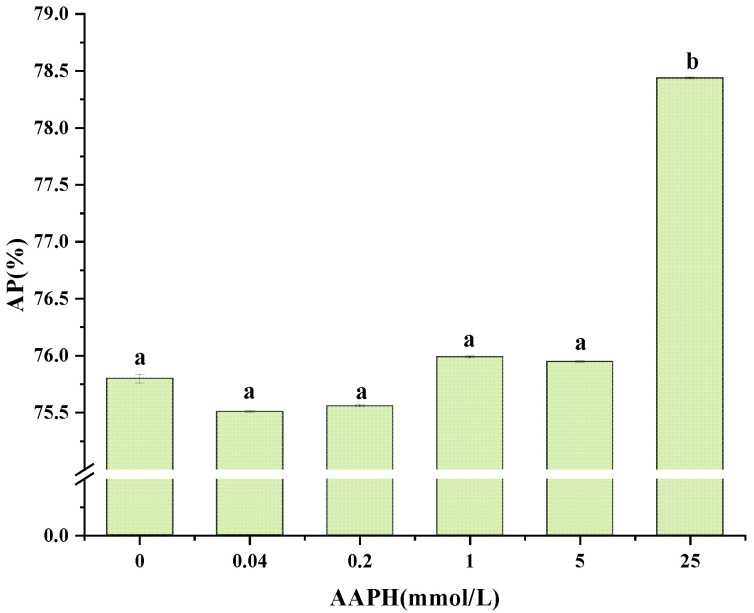
Effect of AAPH oxidation on adsorption rate of adsorbed protein in walnut protein emulsion. Values in a column followed by different letters are significantly different (*p* < 0.05).

Based on Figure 11, the rate at which the oxidized walnut protein emulsion absorbed protein was 75.80% when the AAPH content was 0 mmol/L. The rate of protein adsorption reached a low of 75.56% when the AAPH concentration was 0.2 mmol/L, and reached a maximum of 78.44% when the AAPH concentration was increased to 25 mmol/L. Moderate oxidation in AAPH-oxidized walnut protein emulsions leads to a decrease in protein adsorption rate, which may be attributed to the fact that moderate oxidation opens up the structure of interfacially adsorbed proteins, decreasing the hydrophobicity of the surface of the walnut protein emulsions, and that oil droplets in the emulsions are continuously aggregated, leading to a decrease in adsorption surface area, and, ultimately, proteins are detached from oil–water interfaces, resulting in a decrease in the adsorbed protein rate of the walnut protein emulsions [72]. While the protein adsorption rate began to increase when the AAPH content reached 1 mmol/L, this may be due to the fact that excessive oxidation causes adsorbed proteins to form aggregates which, in turn, form a rigid interfacial film in the interfacial layer of the emulsion, ultimately increasing the interfacial protein adsorption rate of the walnut protein emulsions [73].

### 3.14. SDS-PAGE of Adsorbed Proteins

**Figure 12:**
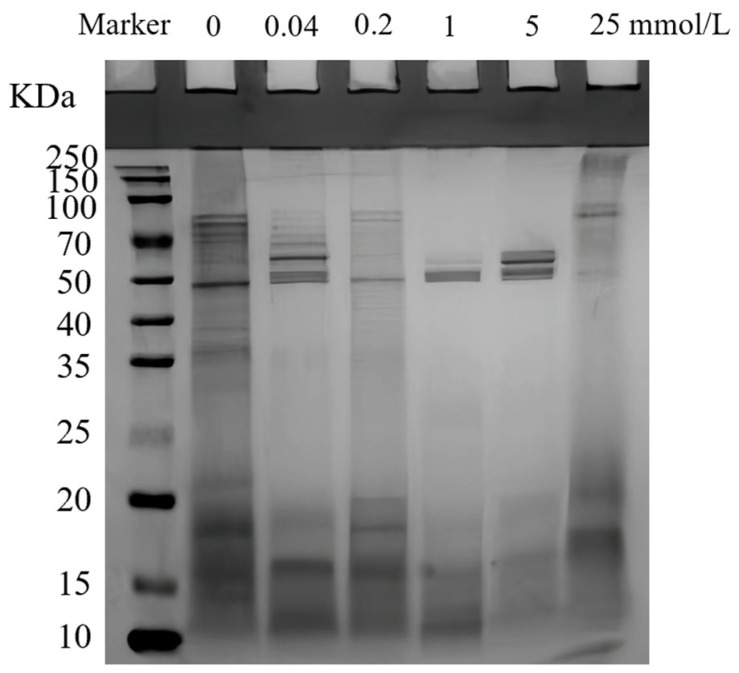
SDS-PAGE of adsorption of walnut protein emulsion by AAPH oxidation.

Polyacrylamide gel electropherograms can be used to examine the impact of oxidation on the subunits of adsorbed proteins in walnut protein emulsions. Figure 12 demonstrates that the molecular weights of the adsorbed proteins were primarily dispersed within the range of 50–100 KDa and below 20 KDa. As the concentration of AAPH increased, the protein that was adsorbed underwent structural unfolding and the large peptide chain was disrupted, resulting in the appearance of aggregates at the top of the gel with varying degrees of color intensity. The intensity of adsorbed protein bands below 20 KDa was highest at an oxidizing concentration of 0.4 mmol/L, suggesting that the protein subunits in this range were more susceptible to oxidation by AAPH. Upon reaching higher concentrations, the gel tip aggregates became the darkest at 25 mmol/, suggesting that β-mercaptoethanol disrupted the disulfide bonds and AAPH oxidation led the adsorbed proteins to form covalent cross-links that were not bound by disulfide [74]. This aligns with the conclusions obtained by Zhu [13]. At a concentration of 1 mmol/L, a reduced number of protein bands with molecular weights ranging from 50–100 KDa were observed, suggesting that oxidation has a considerable impact on the structure of proteins adsorbed in walnut protein emulsions.

### 3.15. Determination of Adsorbed Protein Particle Size

**Figure 13:**
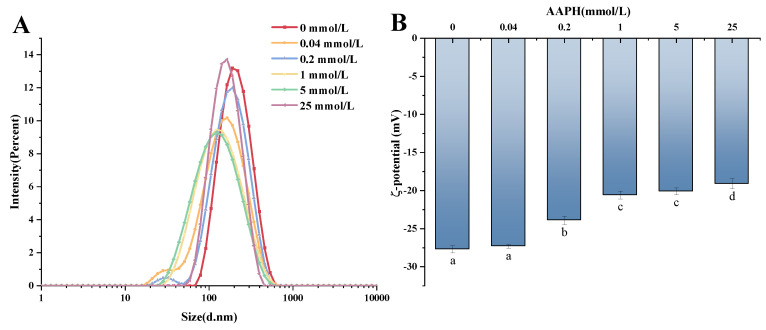
The changes of particle size (**A**) and potential (**B**) of adsorbed protein at the interface of walnut protein emulsion during AAPH oxidation. The values of different letter columns were significantly different (*p* < 0.05).

Figure 13A illustrates that the particle size of adsorbed proteins in walnut protein emulsions initially decreased and then increased as the AAPH concentration increased. This change can be attributed to several factors. Firstly, moderate oxidization led to a decrease in the rate of interfacial protein adsorption, as well as to the unfolding of protein structures and an increase in interfacial membrane viscoelasticity. These effects hindered the formation of aggregates in the emulsions [69]. On the other hand, overoxidization resulted in the formation of aggregates by interfacial adsorbed proteins. This weakened the interfacial membrane viscoelasticity and prevented rigid proteins from forming a viscoelastic network structure. Additionally, the increased Coulombic force likely contributed to the higher rate of interfacial protein adsorption due to the higher content of interfacial adsorbed proteins [73].

### 3.16. Determination of Adsorbent Protein Potential

Figure 13B displays the zeta potential of adsorbed proteins in walnut protein emulsions that were oxidized by AAPH. The figure indicates that the zeta potential gradually increased as the concentration of AAPH oxidation increased. This observation aligns with previous studies on walnut protein emulsions oxidized by AAPH, suggesting that the increase in oxidation concentration causes the point charged amino acids on the protein surface to be enclosed within the proteins [24]. Consequently, this leads to a decrease in the negative charge on the surface of the adsorbed proteins. An AAPH concentration of 0 mmol/L resulted in the highest zeta value of −27.7 mV. The potentials of adsorbed proteins were shown to decrease progressively with the increasing concentration of oxidation. Specifically, the potentials were measured to be −27.3 mV, −23.9 mV, −20.6 mV, −20.1 mV, and −19.1 mV, respectively. This phenomena indicates that, as the concentration increases, the physical stability of the proteins gradually worsens [75].

## 4. Conclusions

Oxidation of emulsions affects the stability and quality of emulsions in storage; therefore, it is extremely important to investigate the effects of protein oxidation on protein emulsions. More precisely, there was an observed rise in the amount of carbonyl groups and a decrease in the amount of sulfhydryl groups and surface hydrophobicity, this change affecting the molecular arrangement and interactions of proteins in walnut protein emulsions. The IR spectroscopy analysis reveals that oxidation has an impact on the secondary structure of walnut protein. Furthermore, as the storage time and AAPH concentration increased, the hydroperoxide value of the emulsions initially rose and then declined. This is due to the conversion of some primary oxidation products into secondary oxidation products during the later stages of oxidation. Consequently, the TBARS content in the emulsions gradually increased, particularly during the late stages of oxidation where it exhibited a noticeable increase (*p* < 0.05). As the concentration of AAPH (0–0.2 mmol/L) increased, the centrifugal force stability of the emulsion decreased. However, the emulsification activity and emulsion stability increased, resulting in a more homogeneous dispersion of protein and oil droplets in the emulsion. From the laser confocal, it was also observed that the protein and oil droplets were dispersed more uniformly in the emulsion. This suggests that moderate oxidation (0–0.2 mmol/L) contributes to the stability of walnut protein emulsions. When the oxidation concentration continued to increase (1–25 mmol/L), the particle size of the emulsion gradually increased, leading to an increase in flocculation and aggregation between droplets, so the emulsifying activity and emulsion stability of the emulsion decreased. The rise in contact angle provided additional evidence that a higher concentration of oxidizing agents had a negative impact on the oxidative stability of the emulsion containing walnut protein. The gel electrophoresis of adsorbed proteins reveals that varying doses of oxidation have distinct effects on the subunit bands, providing evidence that oxidation indeed alters protein structure. Therefore, the stability of a walnut protein emulsion system can be improved by controlling the degree of oxidation of walnut protein. The results of this study provide theoretical basis and technical reference for improving the stability of walnut protein emulsion as well as for the quality of walnut protein products.

## Figures and Tables

**Table 1:** Content of carbonyl group and free sulfhydryl group in walnut protein emulsion oxidized by AAPH at different concentration.

AAPH (mmol/L)	Carbonyl (nmol/mg)	Free Sulfhydryl (nmol/mg)
0	13.45 ± 0.04 ^a^	72.36 ± 0.01 ^d^
0.04	14.48 ± 0.01 ^ab^	69.92 ± 0.05 ^cd^
0.2	15.10 ± 0.01 ^ab^	68.82 ± 0.00 ^c^
1	15.21 ± 0.01 ^b^	65.40 ± 0.01 ^b^
5	15.38 ± 0.01 ^b^	64.54 ± 0.00 ^b^
25	18.83 ± 0.01 ^c^	61.69 ± 0.01 ^a^

Each index of the sample is the average of three determinations, and the data are expressed as mean ± standard error. Different letters in the same column indicate significant differences at the *p* < 0.05 level.

## Data Availability

The original contributions presented in the study are included in the article, further inquiries can be directed to the corresponding author.
